# *Cry1* expression during postnatal development is critical for the establishment of normal circadian period

**DOI:** 10.3389/fnins.2023.1166137

**Published:** 2023-06-14

**Authors:** Aaron E. Schirmer, Vivek Kumar, Andrew Schook, Eun Joo Song, Michael S. Marshall, Joseph S. Takahashi

**Affiliations:** ^1^Department of Neurobiology, Northwestern University, Evanston, IL, United States; ^2^Department of Biology, Northeastern Illinois University, Chicago, IL, United States; ^3^The Jackson Laboratory, Bar Harbor, ME, United States; ^4^Department of Pathology, Massachusetts General Hospital, Boston, MA, United States; ^5^Harvard Medical School, Boston, MA, United States; ^6^Department of Neuroscience, Peter O’Donnell Jr. Brain Institute, University of Texas Southwestern Medical Center, Dallas, TX, United States; ^7^Howard Hughes Medical Institute, University of Texas Southwestern Medical Center, Dallas, TX, United States

**Keywords:** cryptochrome, circadian rhythms, period length, development, gene expression

## Abstract

The mammalian circadian system generates an approximate 24-h rhythm through a complex autoregulatory feedback loop. Four genes, *Period1* (*Per1*), *Period2* (*Per2*), *Cryptochrome1* (*Cry1*), and *Cryptochrome2* (*Cry2*), regulate the negative feedback within this loop. Although these proteins have distinct roles within the core circadian mechanism, their individual functions are poorly understood. Here, we used a tetracycline trans-activator system (tTA) to examine the role of transcriptional oscillations in *Cry1* and *Cry2* in the persistence of circadian activity rhythms. We demonstrate that rhythmic *Cry1* expression is an important regulator of circadian period. We then define a critical period from birth to postnatal day 45 (PN45) where the level of *Cry1* expression is critical for setting the endogenous free running period in the adult animal. Moreover, we show that, although rhythmic *Cry1* expression is important, in animals with disrupted circadian rhythms overexpression of *Cry1* is sufficient to restore normal behavioral periodicity. These findings provide new insights into the roles of the Cryptochrome proteins in circadian rhythmicity and further our understanding of the mammalian circadian clock.

## Introduction

Many biological, physiological, and behavioral processes oscillate with a daily (approximately 24-h) rhythm. These circadian rhythms provide synchrony between an organism and its external environment, allowing the organism to adapt its physiology and behavior to changing environmental conditions (Pittendrigh, 1960). In the mammalian circadian system, light information is detected by specialized ganglion cells in the retina and is then transmitted along the retinal hypothalamic tract to the suprachiasmatic nucleus (SCN) of the hypothalamus (Johnson et al., 1988; Moore et al., 1995; Provencio et al., 2002). The SCN acts as the central circadian pacemaker, regulating circadian rhythms in mammalian behavior and physiology (Low-Zeddies and Takahashi, 2001; Takahashi et al., 2008; Welsh et al., 2010).

Individual cells and groups of cells within the mature SCN have cell-autonomous circadian periods (Welsh et al., 1995; Herzog et al., 1998) and oscillate with different phases (Quintero et al., 2003; Shan et al., 2020). These rhythms are determined by a cell-autonomous molecular clock that is the result of interlocking transcriptional regulatory feedback loops that come together to produce an approximate 24-h cycle (Takahashi, 2017; Cox and Takahashi, 2019). The positive elements in the primary feedback loop are two transcription factors, CLOCK and BMAL1 (Vitaterna et al., 1994; King et al., 1997; Bunger et al., 2000), whereas the negative elements consist of the PER1 and PER2 proteins (members of the PAS protein family) and CRY1 and CRY2 proteins (members of the vitamin B2-based blue light photoreceptor/photolyase protein family; Kume et al., 1999; van der Horst et al., 1999; Zheng et al., 1999; Cermakian et al., 2001). The PER proteins dimerize with the CRY proteins to inhibit the CLOCK and BMAL1 complex, preventing *Per* and *Cry* gene expression (Cox and Takahashi, 2019).

All four of the negative feedback loop proteins are necessary for the core circadian mechanism (Griffin et al., 1999; van der Horst et al., 1999; Vitaterna et al., 1999; Zheng et al., 1999; Bae et al., 2001). However, mice lacking *Cry1* have a short circadian period, while mice lacking *Cry2* have a lengthened period (van der Horst et al., 1999; Vitaterna et al., 1999), suggesting that the CRY proteins have different modes of action within the molecular clock. One limitation of null mouse lines is that they permanently remove genes in an irreversible manner throughout the lifespan of the entire organism. Thus, very little is known about the importance of these proteins in the development of the SCN.

In this study, we used a tetracycline-controlled transactivator (tTA) system to investigate the developmental impact of *Cry1* and *Cry2* expression. We created tg(tetO:*Cry1*) and tg(tetO:*Cry2*) mice, which in combination with pre-existing tg(Scg2:tTA) mice (Hong et al., 2007) allow for inducible, brain-specific overexpression of *Cry1* or *Cry2*, respectively. We first assessed the persistence and maintenance of circadian behavioral rhythms in mice overexpressing *Cry1* or *Cry2* specifically in the brain. Surprisingly, we found that oscillations in *Cry1* and *Cry2* expression are not essential for the persistence of circadian rhythmicity but play an important role in maintaining the period of circadian behavioral rhythms. Next, in order to better understand the role of the molecular clockwork in SCN development, the tTA system was used to overexpress *Cry1* throughout the brain at various points through development. We uncovered a novel role for *Cry1* in the development of free running period and defined a critical period of *Cry1* expression lasting from birth until postnatal day 45 (PN45). Finally, to test the hypothesis that rhythmicity of *Cry1* (not just expression) is necessary for a functional circadian clock, *Cry1* was constitutively expressed in *Cry1/Cry2* null and double null mice to assess its impact on circadian locomotor activity. We found that *Cry1* overexpression in the brain is able to partially rescue behavioral rhythmicity of *Cry1/Cry2* null mice. Together, our findings demonstrate that *Cry1* expression is important for the maintenance of circadian period but is not essential for the persistence of circadian rhythmicity *per se*.

## Materials and methods

### Generation of the tetO:*Cry1* vector

The tetO:*Cry1* vector was constructed using Gateway technology (Invitrogen, Carlsbad, CA). All reactions were performed as described in Invitrogen’s Gateway cloning manual, except at half of the recommended volumes. BP and LR clonase (Invitrogen) were used to transfer a full length *Cry1* cDNA from ATTC (GenBank accession number BCO22174) from pCMV-Sport6 into a pTRE2ppDEST destination vector to facilitate cloning of gateway compatible cDNAs. To do this, the pTRE2 vector (Clontech) was modified by the addition of a gateway destination cassette and four rare restriction endonuclease sites. The gateway destination cassette contained attR sites and positive (Cmr) and negative (ccdB) selection markers required for gateway recombinant cloning. The rare 8 base pair restriction endonucleases PmeI (New England BioLabs, Beverly, MA) and PacI (New England BioLabs) were added into the 5′ end of the tetO sequence and the 3′ end of the poly A by site-directed mutagenesis to allow for removal of the tetO-transgene from the vector backbone for injection into oocytes. All recombinants were transformed into DH5α cells, and positive transformants were selected for on LB + Km 25 μg/mL plates. The presence of the correct fragment in the transformants from both reactions were verified by digestion with BsrG1 (New England BioLabs), followed by gel electrophoresis on a 1% agarose gel in 1X TBE. The vector was then purified on a cesium chloride gradient.

### Generation of the tetO:*Cry2* vector

The tg(tetO:*Cry2*) vector was created from a full length *Cry2* cDNA from ATTC (GenBank accession number BC054794) in the pYX-Asc vector. Due to the incompatibility of the pYX-Asc vector with the Gateway system, the *Cry2* cDNA was first cloned from pYX-Asc into pTRE2pp to convert it to a Gateway compatible vector. pYX-Asc was linearized with AscI (New England BioLabs) and the ends were blunted with T4 DNA polymerase. The fragment was purified on a 1% agarose gel in 1X TBE and the 5.6 kb fragment was extracted using a Qiagen gel extraction kit (Cat. NO. 28706) by the methods described in the QIAquick Spin Handbook (Cat. NO. 28706). Simultaneously, digests were conducted on the pYX-Asc fragment with NotI (New England BioLabs) and the pTRE2pp vector with PvuII (blunt end, New England BioLabs) and NotI. Both of these fragments (a *Cry2* 3.9 kb and a pTRE2 3.8 kb fragment) were gel purified using the Qiagen gel extraction kit. The two purified fragments were then ligated with T4 DNA Ligase (New England BioLabs). The products of the ligation were transformed into DH5α cells and purified on LB + Amp 100 μg/mL plates. The presence of the correct fragments in the transformants and the purification of these fragments were conducted in the same manner as described above for tg(tetO:*Cry1*).

### Creation of tg(tetO:*Cry1*) and tg(tetO:*Cry2*) mice

Using standard transgenic techniques, vectors were linearized with PmeI (New England BioLabs) and microinjected as previously described (Antoch et al., 1997). Transgenic mice were identified by PCR analysis of gDNA using a tetO-specific forward primer (CGCCTGGAGACGCCATCC) and a *Cry1* (ATGAATGGAGGCTGCCGAGG) or *Cry2* (AGGTGTCGTCATGGTTCTCC) specific reverse primer which yield a 400 bp and 748 bp band, respectively. PCR reactions were carried out using 1 μL of extracted gDNA in 11.1 μL water, 0.1 μL of AmpliTaq (Applied Biosystems, Carlsbad, CA), 1.6 μL of 1.25 mM dNTP, 2.0 μL 10X GeneAmp PCR Buffer I (Applied Biosystems), 4.0 μL 5 M Betaine, and 0.1 μL of each primer at a concentration of 100 mM to create a 20 μL reaction. The Thermocycling profile was as follows: 95° for 5 min, followed by 33 cycles of 95° for 15 s, 55° for 30 s, and 72° for 15 s, which was followed by a single extension step of 72° for 2 min. Each individual transgenic mouse resulting from the microinjection was backcrossed to WT C57BL/6 J mice to create the individual hemizygous tetO lines. All mice were maintained in groups of five under standard controlled environmental conditions, with a 12-h light and 12-h dark (12:12 LD) cycle (lights on at 5 am standard time) and free access to food and water unless otherwise stated.

### Conditional overexpression of *Cry* genes driven by tg(Scg2:Tta)

Both tg(tetO:*Cry1*) and tg(tetO:*Cry2*) lines were crossed to a pre-existing driver line to overexpress *Cry1* and *Cry2* in the brain (Hong et al., 2007). Hemizygous tg(Scg2:tTA) mice were crossed with the hemizygous tetO transgenic lines to produce F1 double-transgenic mice, as well as single transgenic and WT controls, as described previously with DTg *Per2* mice (Chen et al., 2009; Supplementary Figure 1A).

### Validation of *Cry* overexpression in double-transgenic mice

Both tg(tetO:*Cry1*) and tg(tetO:*Cry2*) lines were validated by *in situ* hybridization and qPCR to determine expression from the transgene. *In situ* hybridization was conducted as previously described (Sangoram et al., 1998). Briefly, 20 μm coronal sections encompassing the SCN were thaw mounted on gelatin-coated slides. Sections were fixed for 5 min in 4% paraformaldehyde in PBS, treated for 10 min in 0.1 M triethanolamine/acetic anhydride, and then dehydrated through an ethanol series. Slides were hybridized overnight at 47 °C in a hybridization solution composed of 50% formamide, 300 mM NaCl, 10 mM Tris HCL (pH 8.0), 1 mM EDTA, 1X Denhardt’s, 10% dextran sulfate, 10 mM DTT and containing 5 × 10^7^ cpm/mL of the relevant ^33^P-labled probe. The *Cry1* riboprobe was generated from nucleotides 1,015–1,320 of accession number BC022174 while the *Cry2* riboprobe was generated from nucleotides 1,256–1,559 of accession number BC054794. For both probes the nucleotide sequence was PCR amplified and cloned into the pCR 2.1-topo vector (Invitrogen). To prepare the probe, the vector was linearized with HindIII; and the transcription reaction was initiated from the T7 promoter/priming site. The relative expression was quantified using NIH “Image” software version 1.34 s on a Macintosh computer as previously described (Panda et al., 2002).

### Rearing tg(tetO:*Cry1* L1) animals on doxycycline

Hemizygous tg(tetO:*Cry1*) animals were crossed to hemizygous tg(Scg2:tTA) animals to produce double-transgenic, single-transgenic, and WT control animals. All breeding cages were maintained under standard controlled environmental conditions, with a 12:12 LD cycle (lights on at 5 am standard time) with free access to food and water containing 10 μg/mL doxycycline. Doxycycline treatment was started when animals were placed into breeding cages and was continued through conception, gestation, birth, and postnatal development of the pups. Pups were weaned from their mothers at approximately 21 days of age and were maintained in groups of five under the same environmental conditions with free access to food and water containing 10 μg/mL doxycycline until behavior testing.

### Time course of *Cry1* developmental effects

Groups of mice were raised on water and switched to 10 μg/mL doxycycline at points from embryonic day 0 to postnatal day 231. Other groups were reared on 10 μg/mL doxycycline and converted to water from embryonic day 0 to postnatal day 104. Animals were then maintained under the appropriate condition (either on 10 μg/mL doxycycline or on water depending on the paradigm) until the 36th day of the wheel running experiment where they were once again switched to the opposite condition. The timing of the change was based on the date of birth (postnatal day 0).

### Rescue of *Cry1/Cry2* double null animals

*Cry1*−/− / *Cry2*−/− / tg(Scg2:tTA) / tg(tetO:Cry1) animals were produced by systematically crossing the single-transgenic animals to a congenic C57BL/6 J background containing the *Cry1*−/− and *Cry2*−/− alleles. The transactivator line was produced by crossing a hemizygous tg(Scg2:tTA) female to a *Cry1*−/− / *Cry2*−/− male to produce G1 mice. G1s were selected based on PCR amplification of a 500 bp band from genomic DNA (Forward Primer-CAAGTGTATGGCCAGATCTCAA; Reverse Primer-AGACAAGCTTGATGCAAATGAG; 38 cycles with an annealing temperature of 57° followed by a single 72° step (for 5 min) demonstrating the presence of tg(Scg2:tTA). Once *Cry1*± / *Cry2*± / tg(Scg2:tTA) animals were created, they were intercrossed to a siblings of identical genotype to produce *Cry1*−/− / *Cry2*−/− / tg(Scg2:tTA) mice. The operator line was produced by crossing hemizygous tg(tetO:*Cry1*) L1 females with a *Cry1*−/− / *Cry2*−/− male to produce the G1 generation. G1s were selected based on PCR amplification of genomic DNA for the presence of tg(tetO:*Cry1*) to create *Cry1*± / *Cry2*± / tg(tetO:*Cry1*) animals. These animals were intercrossed to siblings of the same genotype to produce the G1F2 generation. *Cry1/2* Genotyping was conducted as previously described (Vitaterna et al., 1999). The transactivator *Cry1*−/− / *Cry2*± / tg(Scg2:tTA) G1F2s were then crossed with the operator *Cry1*−/− / *Cry2*−/− / tg(tetO:Cry1) G1F2s to produce *Cry1*−/− /*Cry2*−/− / tg(Scg2:tTA) / tg(tetO:*Cry1*) mice.

### Circadian activity analysis

Circadian locomotor activity was analyzed for double-transgenic, single-transgenic, and WT-control animals for all tg(tetO:*Cry1*) and tg(tetO:*Cry2*) lines. Wheel-running activity was recorded and analyzed as described previously (Yoo et al., 2004). Briefly, activity (wheel revolutions) was recorded continuously by a PC system ClockLab (Actimetrics, Wilmette, IL) and displayed and analyzed using ClockLab software (Actimetrics, Wilmette, IL). Period was calculated using a Chi-square periodogram (Sokolove and Bushell, 1978) with a six-minute resolution between hours 10 and 36 (ClockLab). The relative power of the circadian component from 18 to 30 h was determined from a normalized Fast Fourier transformation using a Blackman-Harris window (ClockLab). For *Cry1* developmental studies, animals were maintained for 7 days in a 12:12 LD cycle on 10 μg/mL doxycycline followed by constant darkness for 28 days on 10 μg/mL doxycycline and 25 days on water. The free running period was analyzed for days 10–25 (Doxycycline) and 45–60 (H_2_O) respectively. For the rescue experiments, circadian locomotor activity was also analyzed for double-transgenic tg(tetO:*Cry1*) *Cry1/Cry2* double nulls, single-transgenic double nulls, and WT double nulls. Free running period and the relative power of the circadian components were calculated for days 10–25 (H_2_O), days 45–60 (Doxycycline), and days 70–85 (H_2_O). The period and relative power values were compared between groups by ANOVA and Tukey *post hoc* analysis.

## Results

### Overexpression of either *Cry1* or *Cry2* alters circadian behavioral rhythms

**Figure 1:**
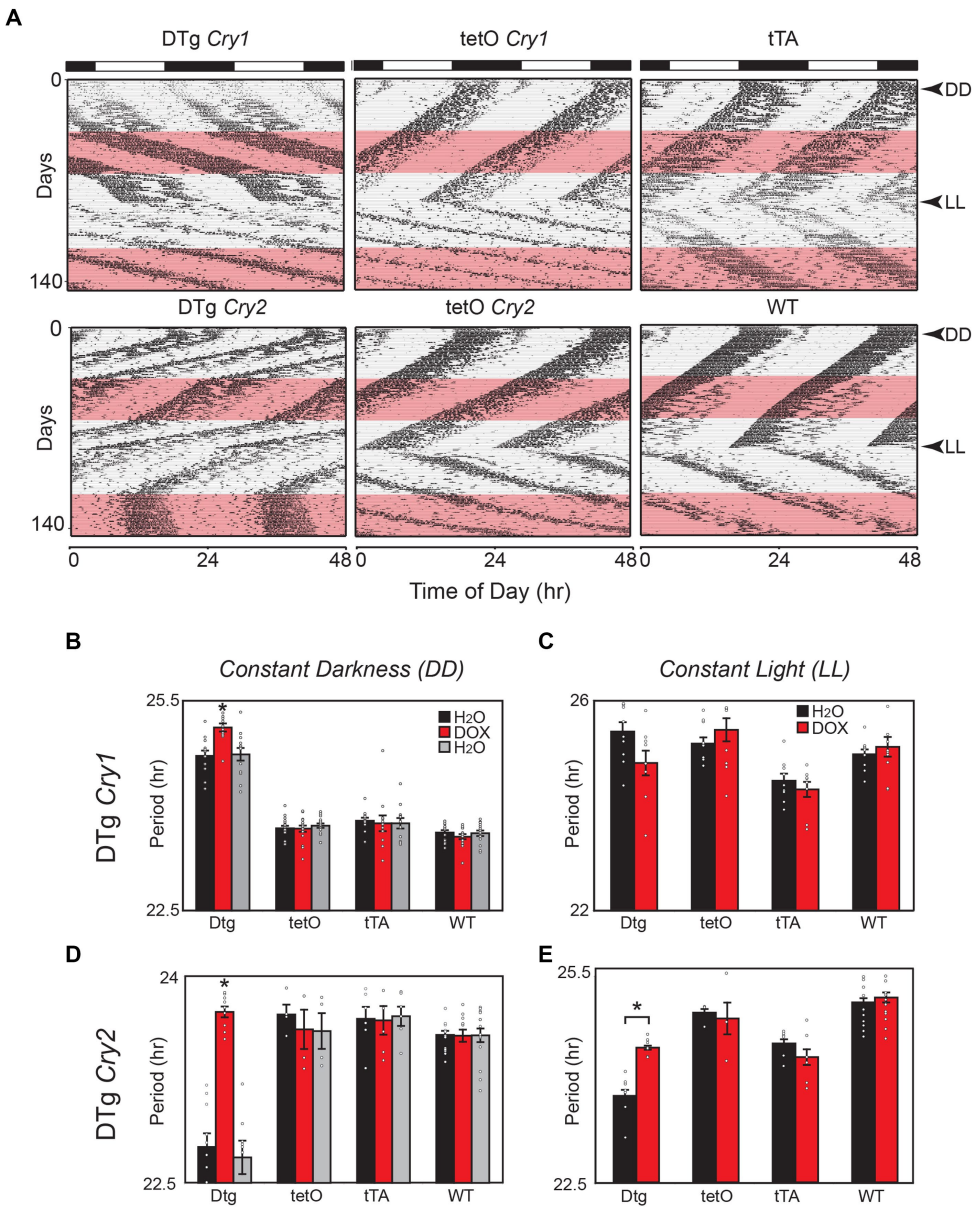
Overexpression of Cry1 and Cry2 in the brain alters circadian behavioral rhythms in mice. **(A)** Representative actograms for double-transgenic (DTg) Cry1 and *Cry2* mice with controls. The pink bar denotes the time of doxycycline administration in constant darkness (DD) and constant light (LL). **(B)** In DD, DTg *Cry1* mice have a clear lengthening in period. Once treated with doxycycline, the period continues to lengthen in DTg *Cry1* mice. DTg *Cry1, n* = 13; tetO *Cry1, n* = 18; tTA *Cry1, n* = 12; WT*, n* = 15; (*p < 0.05). **(C)** In LL, the DTg *Cry1* mice are similar to WT. DTg *Cry1, n* = 10; tetO *Cry1, n* = 10; tTA *Cry1, n* = 12*;* WT*, n* = 15; (* *p* < 0.05). **(D)** In DD, DTg *Cry2* mice display a significantly shorter period. Once treated with doxycycline, the period is returned to WT levels in DTg *Cry2* mice. DTg *Cry2, n* = 10; tetO *Cry2, n* = 4; tTA *Cry2*, *n* = 6; WT, *n* = 15; (*p < 0.05). **(E)** In LL, DTg *Cry2* mice exhibited periods shorter than those seen in WT, while Doxycycline restores the period of DTg *Cry2* mice to levels equivalent with the tetO single-transgenic controls. DTg *Cry2, n* = 4; tetO *Cry2,* n = 5; tTA *Cry2, n* = 12*; WT, n* = 15; (*p < 0.05). Double-transgenic (DTg); Operator only Control (tetO); Transactivator only control (tTA); Wild type (WT).

Both tg(tetO:*Cry1*) double-transgenic (DTg *Cry1*) and tg(tetO:*Cry2*) double-transgenic (DTg *Cry2*) mouse lines demonstrated constitutive overexpression of *Cry1* or *Cry2* in the SCN when compared to single-transgenic and WT controls (Supplementary Figures 1B–E). After this was confirmed, adult DTg *Cry1* and DTg *Cry2* mice were assayed for circadian locomotor rhythms in both constant darkness (DD) and constant light (LL) (Figure 1). DTg *Cry1* animals exhibited a free running period in DD that was significantly longer than WT and single-transgenic controls (Figures 1A,B). This long rhythm was also lower in amplitude than the WT and tetO single-transgenic controls (Average power for DTg *Cry1* = 0.019 ± 0.154, tetO = 0.129 ± 0.009, Scg2 = 0.079 ± 0.019, and WT = 0.150 ± 0.01). There was no additional lengthening of period in DTg *Cry1* mice in response to LL (Figure 1C). In contrast, DTg *Cry2* mice exhibited a free running period that was significantly shorter than WT and single-transgenic controls in both DD and LL (Figures 1D,E).

When the transgene was silenced by treatment with 10 μg/mL of doxycycline, DTg *Cry1* animals exhibited a significant period change and in some cases a phase shift was apparent in DD, although periods remained significantly longer than WT and single-transgenic animals on doxycycline (Figure 1B). Importantly, these changes were reversed by placing mice back on regular water (Figure 1B). In contrast, DTg *Cry2* mice on doxycycline returned to a period comparable to WT and single-transgenic controls (Figure 1D). Interestingly, in LL DTg *Cry2* mice on doxycycline had periods that remained shorter than the tetO single-transgenic and WT controls (Figure 1E). Taken together, these results suggest that oscillations/levels of *Cry1* and *Cry2* are important the maintenance of circadian period; however, they are not necessary for the persistence of circadian activity rhythms. Moreover, these data add to the evidence that *Cry1* and *Cry2* likely have different modes of action within the negative feedback loop of individual cells (Kume et al., 1999; Lee et al., 2001; Van Gelder et al., 2002).

### Developmental *Cry1* overexpression establishes period length in adulthood

**Figure 2:**
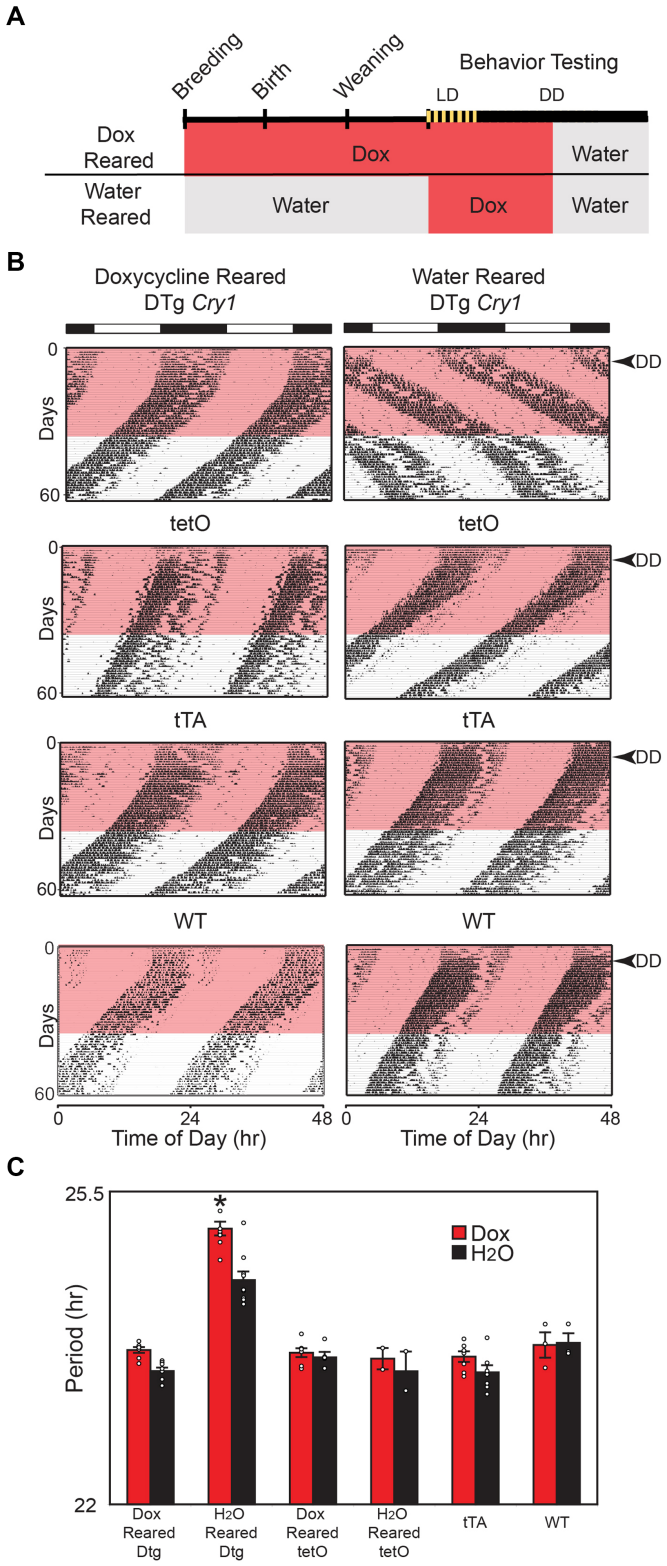
Cry1 overexpression during development is necessary for lengthening of period. **(A)** Schematic of experimental protocol. For the Dox Reared group, Doxycycline (10 μg/mL) was administered in drinking water when mice were placed into breeding cages and was continued through conception, gestation, birth, and postnatal development of pups. Pups were weaned from their mothers at approximately 21 days of age and were maintained on doxycycline up until the 28th day of behavioral testing, when they were switched to water for an additional 25 days. Water reared mice were also administered Doxycycline on days 0–35 of behavioral testing, then switched back to water for the remainder of the experiment. **(B)** Representative actograms of double-transgenic (DTg) *Cry1* mice and controls reared with or without 10 μg/mL doxycycline. The pink bar denotes the time of doxycycline administration during behavioral testing as adults. **(C)** Mean (± SEM) free running period in constant darkness (DD) on and off of doxycycline. The free running period was analyzed for days 10–25 (Dox) and 45–60 (H_2_O). Doxycycline-reared (Dox Reared) DTg *Cry1* mice had a period similar to that of WT and single-transgenic control mice even when treated with doxycycline as adults, while water-reared (H_2_O reared) DTg *Cry1* mice had a lengthened period both with water and doxycycline treatment (*p < 0.05). Double-transgenic (DTg); Operator only Control (tetO); Transactivator only control (tTA); Wild type (WT).

We next asked whether the sustained lengthening of period in the DTg *Cry1* mice in the absence of transgene expression (i.e., on doxycycline) was due to an effect from constitutive *Cry1* expression during development. DTg *Cry1* mice, along with single-transgenic and WT controls, were reared on 10 μg/mL doxycycline (silencing the transgene throughout development); and their locomotor activity rhythms were monitored as adults (Figure 2A). DTg *Cry1* mice reared and kept on doxycycline exhibited a period in DD that was significantly shorter than that of water-reared DTg *Cry1* mice and similar to that of WT and single-transgenic mice (Figures 2B,C). Interestingly, when the doxycycline was removed to allow overexpression of *Cry1* in adulthood, doxycycline reared, DTg *Cry1* mice maintained a period similar to WT (Figures 2B,C). These results suggest that it is the constitutive overexpression of *Cry1* during development that permanently alters the free running period in adult mice.

### *Cry1* expression is required for the postnatal establishment of circadian free-running period

**Figure 3:**
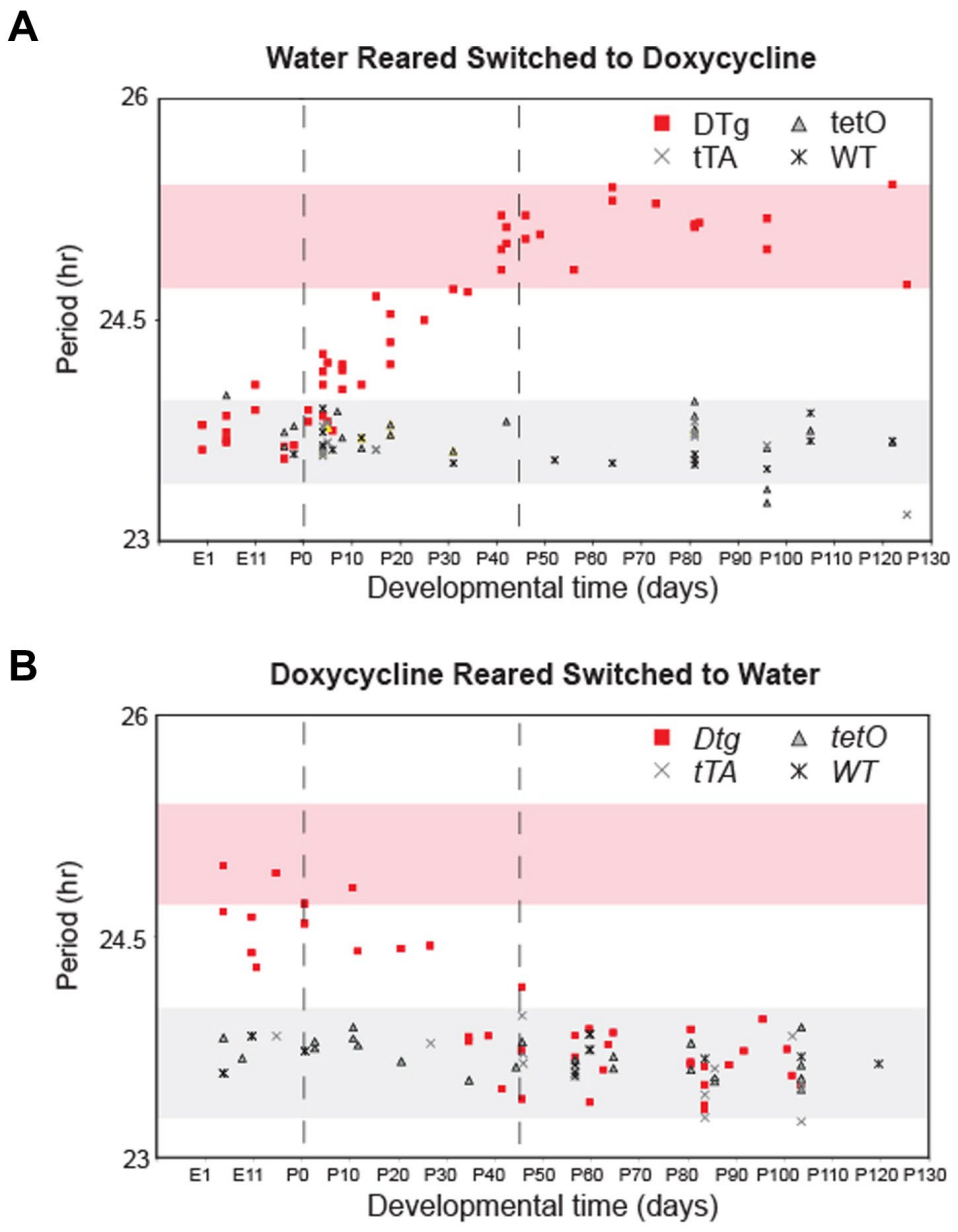
There is a postnatal critical period for Cry1 expression in the establishment of circadian period. Double-transgenic (DTg), single-transgenic, and WT controls were reared on water and converted to 10 μg/mL doxycycline at various developmental time points **(A)** or reared on doxycycline and were converted to water at various developmental time points **(B)**. Each point represents the period of a single adult animal in DD that was switched at a particular developmental time. The x-axis for both graphs is developmental time with PN0 being birth. Pink shading represents a 95% confidence interval around the average period for water reared DTg mice on doxycycline. Gray shading represents the 95% confidence interval around the average period for water reared WT and single-transgenic controls on doxycycline. Points outside of the two 95% confidence intervals are significantly different from water reared DTg mice and controls. Dotted lines indicate boundaries of the critical period for *Cry1* expression when considering data from both experiments.

Next, we sought to define the critical period of the developmental effects of silencing *Cry1*. DTg *Cry1* mice were reared on either water or doxycycline and then were converted to the opposite condition at various times during development. This experimental paradigm allowed for either the inactivation (water to doxycycline) or activation (doxycycline to water) of the *Cry1* transgene during various developmental stages. Circadian locomotor rhythms were then assayed to determine the free running period of the mice in adulthood. DTg mice with the transgene inactivated (water to doxycycline) before birth (PN0) displayed a phenotype similar to that of the doxycycline-reared animals (normal WT phenotype). Conversely, animals for which the transgene was inactivated after PN40 displayed a period similar to the water reared DTg *Cry1* mice (long period phenotype; Figure 3A). In the opposite experiment, DTg mice with the transgene activated (doxycycline to water) before birth (PN0) up until PN45 displayed a period similar to the water reared DTg *Cry1* mice (long period phenotype), whereas DTg mice with the transgene activated after PN45 displayed a phenotype similar to that of the doxycycline-reared animals (normal WT phenotype; Figure 3B). In both the activation and inactivation paradigms, when the switch occurred between PN0 and PN45, a gradient of phenotypes was seen: when *Cry1* expression was higher for longer, the free running period was longer. Thus, combining the data from these two experiments, we can define a critical period from PN0 to PN45 during which the free running period in the adult animal appears to be established.

### *Cry1* overexpression in the brain partially rescues circadian rhythms in arhythmic mice

**Figure 4:**
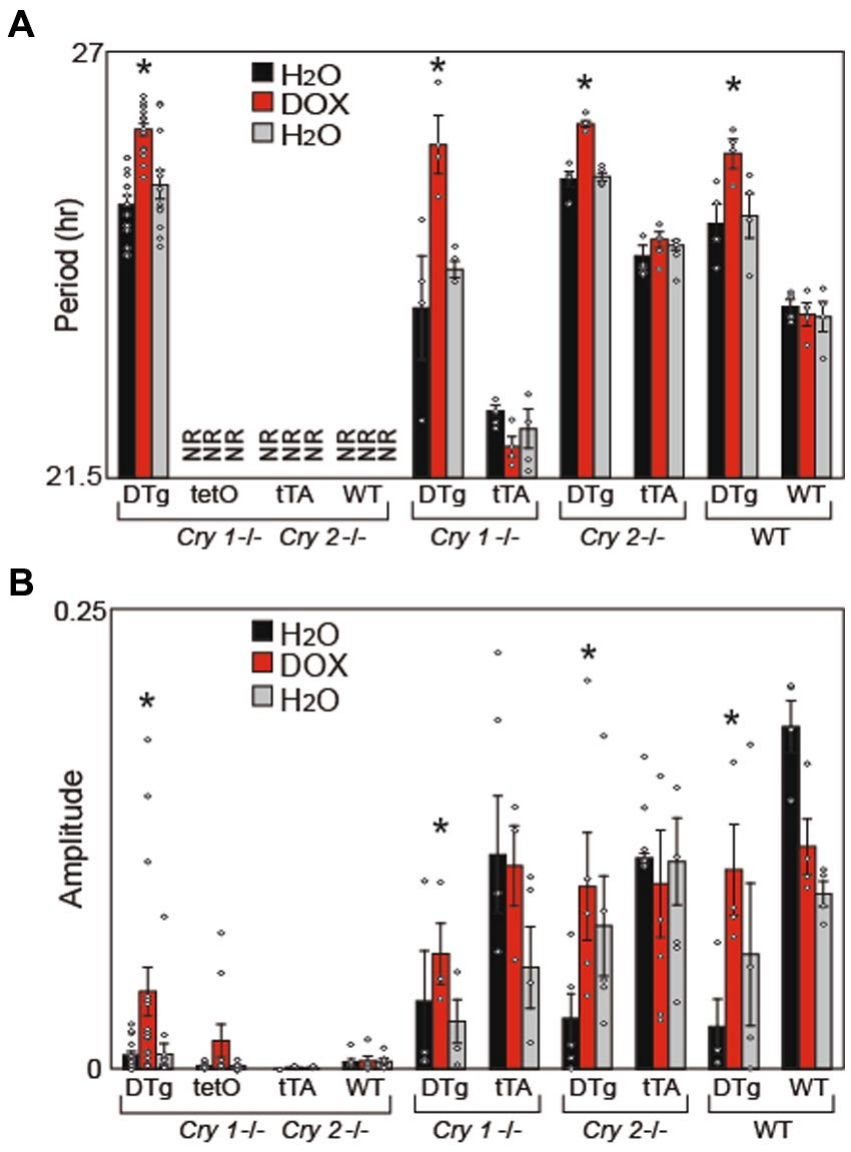
Cry1 Overexpression Rescues Circadian Rhythms in Cry1^−/−^, Cry2^−/−^, and Cry1^−/−^/Cry2^−/−^ Mice: **(A)** Average free running period (Period) and **(B)** FFT power (Amplitude) were calculated for all animals. Period, but not amplitude, was rescued in DTg on a *Cry1*^*−/*−^ and *Cry1*^*−/*−^ / *Cry2*^*−/*−^ background. Values are presented as mean ± SEM (*p < 0.05); NR = non-rhythmic mice (from which we are unable to calculate a period). Double-transgenic (DTg); Operator only Control (tetO); Transactivator only control (tTA); Wild type (WT). DTg *Cry1*^*−/*−^ / *Cry2*^*−/*−^, *n* = 20; tetO *Cry1*^*−/*−^ / *Cry2*^*−/*−^, *n* = 10; tTA *Cry1*^*−/*−^ / *Cry2*^*−/*−^, *n* = 3; WT *Cry1*^*−/*−^ / *Cry2*^*−/*−^, *n* = 7; DTg *Cry1*^*−/*−^, *n* = 4; WT *Cry1*^*−/*−^, *n* = 4; DTg *Cry2*^*−/*−^, *n* = 5; WT *Cry2*^*−/*−^, *n* = 3; DTg WT*, n* = 4; WT WT, *n* = 4.

Because the transgenes we utilized overexpress *Cry1* or *Cry2*, we assumed that the normal rhythmic expression of these genes is disrupted. Thus, to better understand whether rhythmicity of *Cry1* (and not just expression) is required to establish circadian rhythms, *Cry1* was constitutively expressed (tg(tetO:*Cry1*) driven by tg(Scg2:tTA)) on a *Cry1*/*Cry2* double null background as well as on individual *Cry1*^−/−^ and *Cry2*^−/−^ background; and locomotor activity rhythms were assessed. Constitutive *Cry1* expression was found to partially rescue circadian locomotor activity rhythms on both the *Cry1*/*Cry2* double null and individual *Cry1*^−/−^ mice, with period lengths comparable to WT mice (Figure 4A; Supplementary Figure 2). However, the amplitude of these rhythms never returned to WT levels (Figure 4B). Surprisingly, when constitutive *Cry1* expression was silenced with 10 μg/mL doxycycline mice on a *Cry1*/*Cry2* double null background, these mice showed lengthening of period in a manner similar to that seen in the DTg *Cry1* mice on a WT background (Figure 4A). A loss of circadian rhythmicity (as would be expected in single-transgenic and *Cry1*/*Cry2* double null mice) was never seen. Together with our other results, these findings strongly suggest that rhythmicity in *Cry1* expression is not necessary, but rather the overall level of *Cry1* expression is what drives period length.

## Discussion

In this study, *Cry1* and *Cry2* were constitutively overexpressed in the brain leading to animals that were rhythmic, but with altered periods. The constitutive overexpression of *Cry1* led to a lengthening of free running period, while constitutive overexpression of *Cry2* led to a shortening of free running period. These results make sense in light of the opposite phenotypes seen in *Cry1* and *Cry2* knockout mice (van der Horst et al., 1999; Vitaterna et al., 1999), but are none-the-less surprising, since the overexpression of these genes precludes the normal cycling of *Cry1* or *Cry2* gene expression. Interestingly, when *Cry2* overexpression was silenced with treatment of 10 μg/mL of doxycycline, the mice exhibited a period comparable to WT, while this same treatment in *Cry1* DTg mice continued to lengthen the free running period, suggesting a developmental role for *Cry1* in the establishment of circadian period. To test this hypothesis, we reared DTg *Cry1* mice from conception on doxycycline, and found that the constitutive overexpression of *Cry1* during development led to permanent alterations in free running period. Subsequently, we defined a critical period from birth to postnatal day 45 where the expression of *Cry1* permanently altered circadian period in adult mice.

There are several mechanisms that could explain how and why this developmental change is occurring. First, it possible that inactivity of the tetO transgene during development can lead to their functional silencing (Zhu et al., 2007). However, we show that activation as well as inactivation of the transgene during development can lead to similar phenotypic effects that both fall within the same critical period, suggesting that the effects are not due to functional silencing or transgene inactivation.

Another possible mechanism could be through epigenetic modifications. Rhythmic changes in histone acetylation at circadian clock gene promoters are associated with chromatin modifications (Etchegaray et al., 2003; Doi et al., 2006; Ripperger and Schibler, 2006; Koike et al., 2012). Interestingly, CRY1 has been found to interact with a histone acetyltransferase to alter CLOCK/BMAL1-mediated transcription (Etchegaray et al., 2003), potentially providing a direct mechanism for long-term CRY1 effects. Future studies using tissues derived from these mouse models could help elucidate the molecular impacts of developmental *Cry1* expression.

Although preliminary data (not shown) suggest that the gross morphology of the SCN is normal in our DTg mice, it is also possible that the cellular phenotypes in the SCN are altered. In addition, the level or oscillation of *Cry1* could developmentally alter the synaptic connectivity and coupling in the SCN. Heterogeneous cellular phenotypes are integrated in the adult SCN, decreasing cycle-to-cycle variability in order to determine the generation and expression of circadian rhythms (Low-Zeddies and Takahashi, 2001; Herzog et al., 2004). Moreover, loss of function in clock genes has the ability to impact neural firing and synchrony in the SCN (Schwartz et al., 1987; Albus et al., 2002; Ono et al., 2013; Shan et al., 2020). Importantly, oscillations in fetal SCN neurons are known to be present as early as embryonic day 17, before synaptic connections are developed (Reppert and Schwartz, 1984; Speh and Moore, 1993; Shimomura et al., 2001; Carmona-Alcocer et al., 2018), suggesting they may play an important role in synaptic connectivity. Theoretically, alteration of *Cry1* expression and oscillations in fetal SCN neurons during the critical period could alter the firing patterns and therefore change the synaptic connectivity and neural representation of period within the adult SCN (Sumova et al., 2012, Ono et al., 2013). Our data are consistent with this theory because the critical developmental period matches the peak of synaptogenesis in the maturing SCN (Honma, 2020). However, these synaptic changes are likely subtle, as we see no evidence gross alterations in SCN morphology (data not shown).

In other organisms, the rhythmic requirements of the negative elements of the primary feedback loop have been elucidated by constitutively expressing these negative elements on a null background (Aronson et al., 1994; Edwards et al., 2016). *Cry1*/*Cry2* double null mice lack circadian rhythmicity in constant darkness, while individual *Cry1* or *Cry2* null mice are rhythmic but have abnormal periodicity (van der Horst et al., 1999; Vitaterna et al., 1999; Bae et al., 2001). The results of our experiments demonstrate that constitutive, brain-specific expression of *Cry1* can partially rescue circadian locomotor rhythms on a *Cry1*/*Cry2* double null background and suggest that oscillations in *Cry1* are not necessary for the maintenance of circadian locomotor period. Although we did not examine CRY1 protein expression, our findings are consistent with recent experiments which showed that overexpression of CRY1 protein rescues SCN rhythms in *Cry1*/*Cry2* null mice (McManus et al., 2022). There are also similar findings with the constitutive expression of CRY1 in Rat-1 fibroblasts (Fan et al., 2007; Yamanaka et al., 2007) and in the liver of mice (Chen et al., 2009). Interestingly, the amplitude of the rhythms was not rescued back to WT, therefore oscillations in *Cry1* expression may be necessary for the maintenance of circadian amplitude.

Finally, both CRY1 and CRY2 have been shown to repress CLOCK/BMAL1 in distinct ways (Kume et al., 1999; Lee et al., 2001; Van Gelder et al., 2002). CRY1 is thought to be a potent inhibitor of the positive feedback loop by directly interacting with the CLOCK/BMAL1 complex (Griffin et al., 1999; Kume et al., 1999; Shearman et al., 2000); although, it may require PER to act as a scaffolding protein to facilitate this interaction (Chen et al., 2009) as it has been shown to impact nuclear shuttling of PER (Smyllie et al., 2022). In contrast, CRY2 is thought to associate strongly with the CLOCK protein (Griffin et al., 1999). Our results suggest a fine-tuning role of *Cry2* in the negative feedback loop (Griffin et al., 1999). In particular, our finding of an effect of LL in DTg *Cry2* mice suggests an alteration of the phase response curve, leading to the intriguing possibility that *Cry2* plays a role in mediating the response to light.

In summary, our data support a novel, developmental role for *Cry1* expression in the SCN and also call into question the necessity of *Cry1* rhythmic expression after a critical period in development. These findings further our understanding of the mechanisms behind the generation of circadian rhythmicity. However, more research is needed to further determine how the various molecular feedback loops work together to generate rhythmicity, as well as how these feedback loops are interacting with rhythm generating components such as neural network oscillations.

## Data availability statement

The original contributions presented in the study are included in the article/Supplementary material, further inquiries can be directed to the corresponding author.

## Ethics statement

The animal study was reviewed and approved by Institutional Animal Care and Use Committee, Northwestern University.

## Author contributions

AES planned and performed behavior experiments, analyzed data, made figures and wrote the manuscript. VK provided technical assistance with cloning. AS performed *in situ* hybridization experiments. EJS performed microinjection for transgenic mouse lines. MSM performed mouse colony maintenance and collected data. JST planned and helped with data analysis and writing of the manuscript. All authors contributed to the article and approved the submitted version.

## Funding

This work was supported by a Silvio O. Conte Center NIH Grant P50 MH074924 to JST. JST is an Investigator in the Howard Hughes Medical Institute.

## Conflict of interest

The authors declare that the research was conducted in the absence of any commercial or financial relationships that could be construed as a potential conflict of interest.

## Publisher’s note

All claims expressed in this article are solely those of the authors and do not necessarily represent those of their affiliated organizations, or those of the publisher, the editors and the reviewers. Any product that may be evaluated in this article, or claim that may be made by its manufacturer, is not guaranteed or endorsed by the publisher.
