# Supplementary figures and images for: Cry1 expression during postnatal development is critical for the establishment of normal circadian period
Source: Front Neurosci. 2023 Jun 14;17:1166137. doi: 10.3389/fnins.2023.1166137 (PMC10300422; doi:10.3389/fnins.2023.1166137)

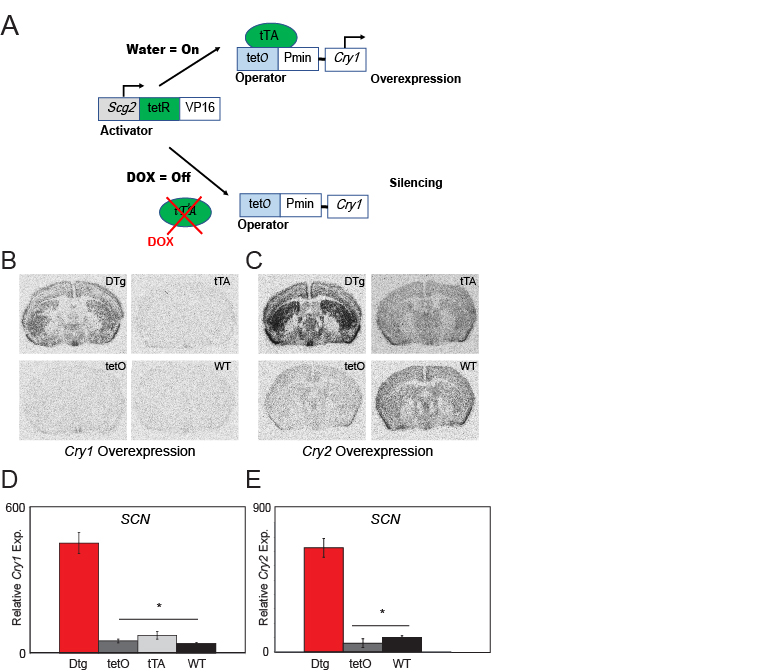

Supplement: Supplementary file 1 [file Image_1.JPEG]

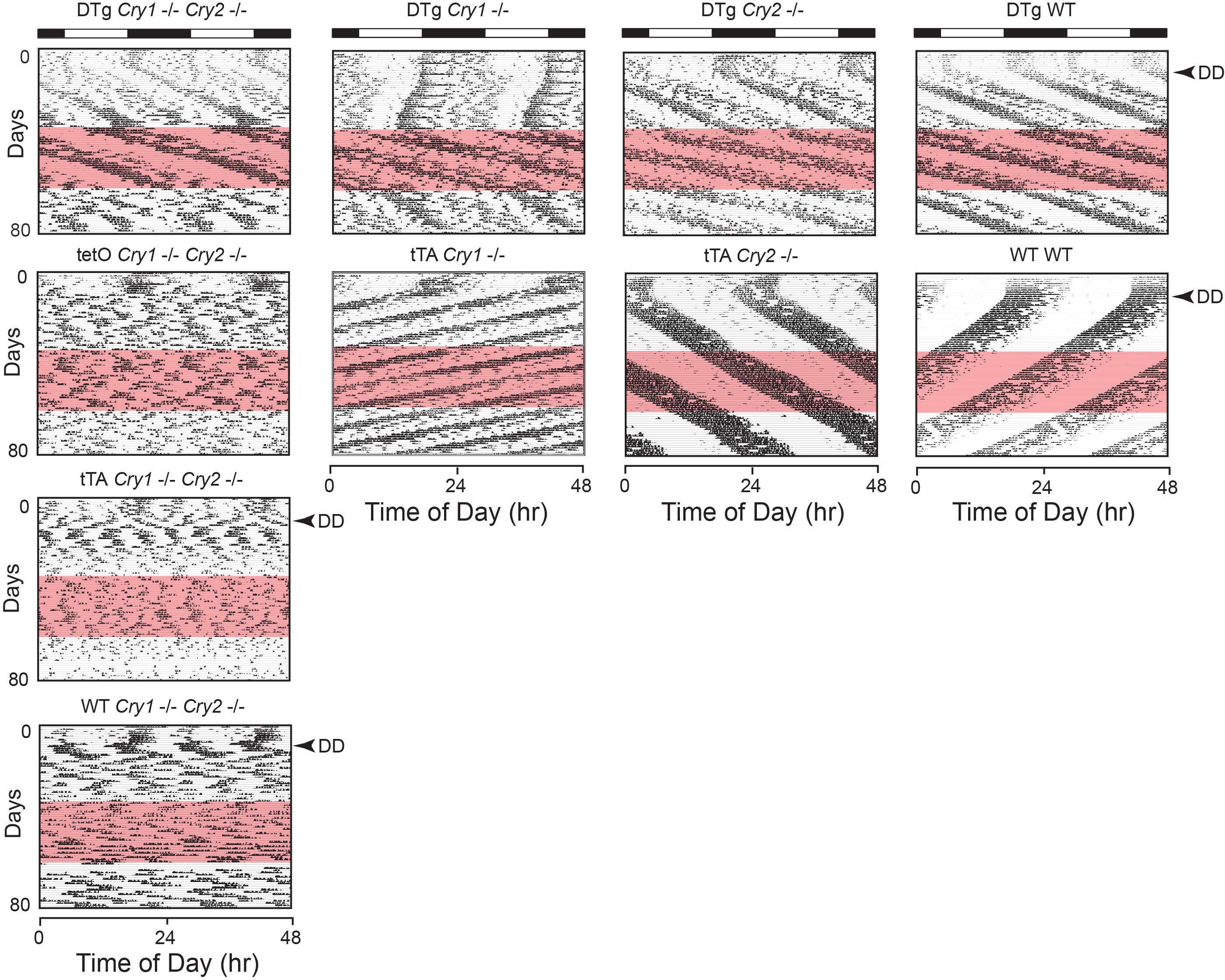

Supplement: Supplementary file 2 [file Image_2.jpg]
